# The Additive Manufacturing Approach to Polydimethylsiloxane (PDMS) Microfluidic Devices: Review and Future Directions

**DOI:** 10.3390/polym15081926

**Published:** 2023-04-18

**Authors:** Anthony Tony, Ildiko Badea, Chun Yang, Yuyi Liu, Garth Wells, Kemin Wang, Ruixue Yin, Hongbo Zhang, Wenjun Zhang

**Affiliations:** 1 Department of Mechanical Engineering, University of Saskatchewan, Saskatoon, SK S7N 5A9, Canada; ant359@mail.usask.ca (A.T.); chy416@mail.usask.ca (C.Y.); yul994@mail.usask.ca (Y.L.); 2 College of Pharmacy and Nutrition, University of Saskatchewan, Saskatoon, SK S7N 5E5, Canada; ildiko.badea@usask.ca; 3 Synchrotron Laboratory for Micro and Nano Devices (SyLMAND), Canadian Light Source, Saskatoon, SK S7N 2V3, Canada; garth.wells@lightsource.ca; 4 School of Mechatronics and Automation, Shanghai University, Shanghai 200444, China; wangkemin@shu.edu.cn; 5 School of Mechanical and Power Engineering, East China University of Science and Technology, Shanghai 200237, China; yinruixue@ecust.edu.cn

**Keywords:** polydimethylsiloxane, 3D printing, microfabrication, microfluidics, post treatment

## Abstract

This paper presents a comprehensive review of the literature for fabricating PDMS microfluidic devices by employing additive manufacturing (AM) processes. AM processes for PDMS microfluidic devices are first classified into (i) the direct printing approach and (ii) the indirect printing approach. The scope of the review covers both approaches, though the focus is on the printed mold approach, which is a kind of the so-called replica mold approach or soft lithography approach. This approach is, in essence, casting PDMS materials with the mold which is printed. The paper also includes our on-going effort on the printed mold approach. The main contribution of this paper is the identification of knowledge gaps and elaboration of future work toward closing the knowledge gaps in fabrication of PDMS microfluidic devices. The second contribution is the development of a novel classification of AM processes from design thinking. There is also a contribution in clarifying confusion in the literature regarding the soft lithography technique; this classification has provided a consistent ontology in the sub-field of the fabrication of microfluidic devices involving AM processes.

## 1. Introduction

Microfluidics is a discipline of circuitry devices, which studies the manipulation of fluids in a logical manner [1,2,3,4]. Fluid flow in a microfluidic device is laminar due to the size of the channel, and the flow may be a mix of flows to increase the function of such devices [2,5,6]. Fast response during experimentation can be achieved and visualized via small spaces along with their corresponding properties, including short inflow distance and low diffusion. Also, a small volume means less consumption of reagents and energy, and an easy integration of different domains (e.g., micro-optics in between two microfluid flows) can lead to fast processing and efficient in situ testing [7].

Microfluidic devices made of polydimethylsiloxane (PDMS) are widely used in biomedical science and technology [8,9,10,11,12,13] due to the biocompatibility of PDMS. Other important properties with PDMS are optical transparency, thermal stability, curability at room temperature, resistance to UV radiation, durability, hydrophobicity, physiological inertness, high permeability to gases, and anti-friction. These properties consolidate potential uses of PDMS microfluidic devices in biomedical systems and facilitate building of such devices [14,15,16]. In addition, the surface of PDMS is modifiable to change its physical, biological, and chemical properties to render to some unique applications with PDMS microfluidic devices [17,18,19,20,21]. However, PDMS also has some limitations, e.g., swelling, which causes significant distortions in geometry, and these limitations need to be carefully considered in printing of PDMS [13,22,23,24]. This paper focuses on PDMS microfluidic devices for biomedical applications.

Microfluidic devices are not conventional MEMS, which usually require that their overall size is less than 100 microns. In microfluidic devices, only their channels or holes are less than 100 microns, but their overall size is usually in the mm range [1,2,3]. The lithography (including soft lithography) approach may not be quite suitable to fabricating microfluidic devices; instead, additive manufacturing (AM) technology has promising potential to build microfluidic devices, especially PDMS microfluidic devices. This paper focuses on PDMS microfluidic devices by AM processes.

The requirements for building a PDMS microfluidic device include: (1) 3D structures with channels (**R1**), (2) micron scales of the channel size as well as holes (**R2**), (3) accuracies in the shape and geometry of channels as well as pores (about 1–5 microns) (**R3**), (4) proper surface features of the channel (especially for easy assembly or bonding of multiple PDMS parts) (**R4**), (5) mass production (**R5**), and (6) production rate (**R6**).

This paper presents a critical review of the AM approach to PDMS microfluidic devices, aiming to identify knowledge gaps and to suggest future work to close these gaps. Specifically, Section 2 presents a unique classification of AM processes based on our observation that there is much confusion about the understanding of the AM process, e.g., the relationship of the AM and 3D printing. With the background presented in Section 2, Section 3 presents a review and analysis of AM processes for PDMS microfluidic devices, including our ongoing work. Section 4 concludes the paper by summarizing the knowledge gaps and suggesting future work to close the gaps. There is also a summary of the contributions of this paper in the last section.

## 2. The AM Process along with Its Classification

Applying an approach called ‘design thinking’ to any process or system [25], any AM process needs to fulfill two functions: F1: create one layer of solid; F2: glue two layers of solids. It is noted that the processes for F1 and F2 may be simultaneously carried out. For instance, while one layer may not completely form a solid, a subsequent layer is spread on top of the first layer, and both layers become solids simultaneously. A particular AM process can thus be divided into two sub-processes to fulfill F1 by Sub-process 1 and F2 by Sub-process 2, respectively. Each process transforms material, energy, and/or data [26]. As such, each AM can be classified in terms of (i) the starting statuses of materials and (ii) the principles of processes (Sub-process 1, Sub-process 2) to achieve F1 and F2, respectively (see Table 1, where the process name and equipment name, if any, are included). Special attention is given to fused deposition modeling (FDM) (in Table 1), which was also called 3D printing in the early days when FDM was developed. According to Table 1, 3D printing is a kind of AM. However, today, the term ‘3D printing’ has become a nickname for all AM processes in the literature. Therefore, in this paper, AM and 3D printing are used interchangeably unless otherwise their difference is stated explicitly. Care must also be taken that there is effort on developing ISO standard for classification of AM, e.g., ISO ASTM 52900. However, in our view, the criteria for rendering the classification of AM in the ISO standard are different from ours in this paper; in the ISO standard, the criteria appear to be more on how the material is delivered on the substrate, which may be more suitable to industry but not to science of AM. Several specific remarks are further made and discussed below.

**Remark** **1.** *FDM processes are simple, cost effective, and common; commercially available in the market; and they work on the principle of depositing molten material layer by layer. FDM supports a variety of thermosetting and thermoplastic materials, such as acrylonitrile butadiene styrene (ABS), polylactic acid (PLA), and polyethylene terephthalate (PET) [27]. Microfluidic devices made with FDM have several drawbacks, such as the irregular channel shape, dimension inaccuracy, low optical transparency, poor reliability, and limited air permeability [28]. FDM processes also have some difficulty in creating products with the surface characteristics of biocompatibility, which is crucial to chemical and biological applications. However, in recent years, some enhancements to FDM were reported in literature, e.g., [29,30,31], some of which have potential to be used to print PDMS microfluidic devices [30].*

**Remark** **2.** *In the literature, there is a notion called **soft lithography**, which was originally proposed in [21] to fabricate micro-systems made of polymers (such as PDMS). According to [32], the soft lithography process was defined as producing polymer products, e.g., PDMS products, by using the lithography approach to build a mold. Usually, the mold in the context of soft lithography is made of hard materials such as silicon-based materials. Such a soft lithograph method has difficulty in fabricating rounded [33,34], non-planar [35], sloped [36], and tapered [37] cross-sectional channels in microfluidic devices. Recently, it has been shown that the mold can be made by other fabrication approaches, e.g., micro-milling, AM, etc. Nonetheless, the name ‘soft lithography’ is kept for all of these methods (including the 3D printing method) to make a mold. This paper is focused on making molds using the 3D printing method. In the literature, the general term “**printed mold**” [38] may also be used for this approach. Hereafter in this paper, the terms ‘printed mold’ and ‘soft lithography’ are used interchangeably unless otherwise their difference is mentioned. The present paper is not only about the printed mold approach but also about other printing approaches to fabricating PDMS microfluidic devices.*

**Remark** **3.** *In general, FDM can produce a rough surface (10.97 μm) compared with Polyjet (0.99 μm) and digital light processing (DLP)-SLA (0.35 μm). Therefore, FDM is usually used for fabricating microfluidic devices for the task of mixing two or more fluids [39], while DLP-SLA is used for fabricating microfluidic devices suitable for precise flow control applications. Further, Polyjet is usually used for fabricating microfluidic devices suitable to cell culture or droplet generators, where splitting of multiple flows is avoided. For specific comparisons of different AM processes in terms of the material used, we refer to [40].*

## 3. Printing of PDMS Microfluidic Devices

**Figure 1:**
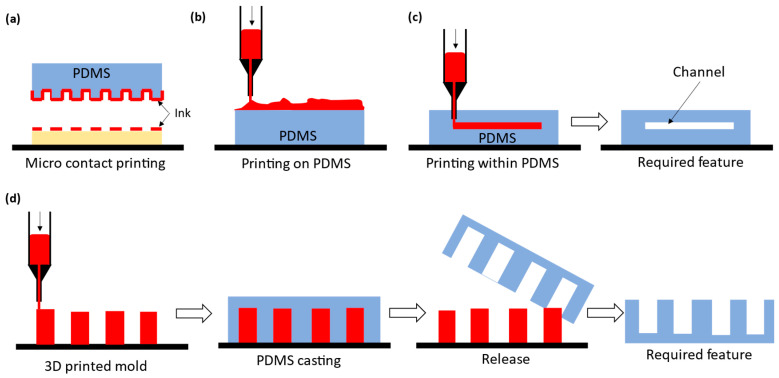
The classification of the indirect PDMS printing techniques. (**a**) Surface patterning technique, commonly known as microcontact printing, using PDMS as a stamp mold for transferring the ink pattern, (**b**) printing on PDMS, (**c**) printing within PDMS and subsequent selective removal of the printed part giving the required feature, and (**d**) printed mold PDMS.

**Figure 2:**
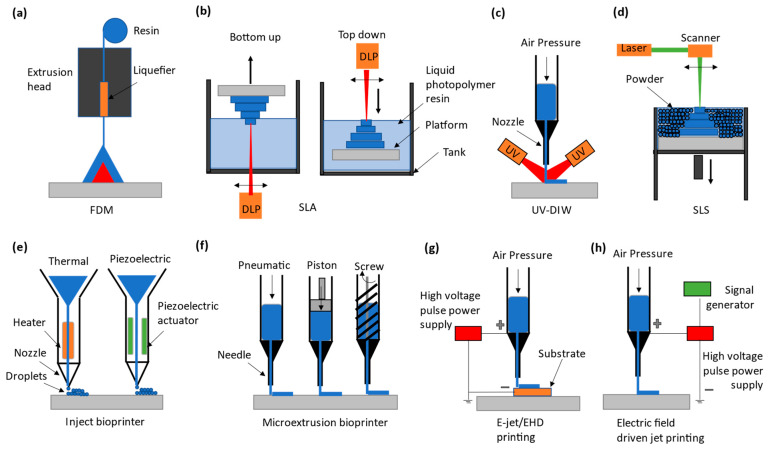
The classification of the direct PDMS printing techniques. (**a**) FDM extrudes solid thermoplastic PDMS filament and melts and deposits using a heated nozzle [41,42]. (**b**) SLA or vat polymerization, showing bottom-up and top-down approaches to printing PDMS using a UV laser or DLP-UV light [43,44,45,46]. (**c**) DIW, in which PDMS-based liquid ink flows through a nozzle and solidifies upon UV exposure after deposition [47]. (**d**) SLS uses irradiation via a laser which creates localized melting inside a bed of solid powder PDMS [48]. (**e**) Inkjet printing ejects small droplets of PDMS which solidify when exposed to light or heat [49,50]. (**f**) Micro extrusion printers dispense liquid PDMS resin, either using pneumatic, piston, or screw actuation through a needle [51,52,53,54]. (**g**) Electrohydrodynamic (EHD) or E-jet printing provides very fine, even submicron-scale, PDMS printing driven by an electric field [55]. (**h**) Electric-field-driven (EFD) printing, which closely resembles EHD printing, does not require conductive substrates while printing [56].

As suggested from Remark 2 in Section 2, great attention is paid to involving AM in the fabrication of microfluidic devices, especially those made by PDMS, aimed for biological applications. The AM approach to microfluidic devices can be classified into two kinds: direct printing and indirect printing. Indirect printing means that the PDMS product is completely printed out but produced by involving other manufacturing approaches, e.g., casting. Figure 1 shows some developments in the literature on the indirect printing approach. It can be seen from Figure 1 that there are four situations with the indirect printing approach to PDMS microfluidic devices. The first three situations, (a) to (c), refer to situations where (a) PDMS is a tool to make another microfluidic device, (b) PDMS is part of the final product with the printed part added on it, and (c) PDMS is the final product, where channels are printed by another material and then this material is vaporized. In Figure 1d, a mold needs to be made first, and then the PDMS is cast with the mold. Direct printing means that the PDMS product is completely printed; see Figure 2, where several direct printing processes are illustrated [33,34,35,36]. It is worth mentioning that Figure 2e–h refer to the process of LDM with differences being how the material is delivered to the board or substrate. In the following, the selected processes of indirect printing (Section 3.1, Section 3.2, Section 3.3 and Section 3.4) and direct printing (Section 3.5) are reviewed.

### 3.1. Indirect PDMS Printing: Micro Contact Printing

Functionalization of certain chemical or biological samples onto PDMS surfaces (Figure 1a) can be done using micro contact printing with PDMS. In this process, PDMS is replicated from a master mold, acting like a stamp after curing. Applications of this idea with PDMS range from protein printing, pattern of hydrophobic alkanethiols [57], arrangement of patterned neuronal cells [58], chemical synthesis [59], embedding carbon nanotube composites [60], and bilayer polymer solar cells [61]. Printing processes of this kind create essentially a 2D feature, as the third dimension or the height of the printed layers is negligible (nanometer). Moreover, the aspect ratio (width to height) of the printable layers is very low. Clearly, this approach poorly meets R1 for PDMS microfluidic devices (see the discussion in Section 1).

### 3.2. Indirect PDMS Printing: Printing of Materials on PDMS

Printing on top of PDMS (Figure 1b) is an emerging sub-field due to growing demand for high-performance devices such as stretchable, wearable, electronic skins, and soft conductive materials [38,62]. Traditionally, the following processes are used to add materials on top of PDMS: chemical reduction, evaporation and sputtering, screen printing, lift-off, pattern transfer, and soft lithography [63]. These processes are time consuming, expensive, and involve complex protocols. A more effective process is AM. The inkjet printing of conductive materials onto PDMS is one of the simplest processes to add materials onto PDMS. The coalesced inkjet droplets tend to become larger droplets due to the driving force and low adhesion of the PDMS surface [62,64]. Printing of silver nanoparticle (NP)-based ink on top of a pre-stretched PDMS enables the possibility of making the circuit stretchable and more adaptable [65]. Patterning of metals on PDMS for control and detection are also reported [62]. This can be integrated with roll-to-roll (R2R) screen printing, a type of lamination technique, to achieve a high-dimensional precision PDMS microfluidic device with multi-functionalities [66].

Printing of materials, particularly conductive metals or electrodes, on PDMS has some challenges due to the low surface energy (~25 ± 4 mN/m) and hydrophobic property (static contact angle of 109 ± 3°) of PDMS [64,67]. Therefore, it is difficult to meet the requirements for PDMS microfluidic devices (R3 and R4), as described in Section 1. Proper modification or treatment of the PDMS surface may therefore be needed to make this approach work, although this increases the complexity of the approach. Moreover, this approach is not favorable to meeting R5 for PDMS microfluidic devices (i.e., mass production).

### 3.3. Indirect Printing: Printing within PDMS

Printing within PDMS is done while PDMS is in a liquid viscous form (Figure 1c). The printed part along with PDMS is cured, and then the printed part is selectively dissolved and melted away to achieve the required feature, as shown in Figure 1c. The entire process involves both subtractive manufacturing and additive manufacturing processes [68,69], and therefore, the entire process is called suspended liquid subtractive lithography (SLSL). It is noted that in the literature, SLSL may be confused with the embedded three-dimensional (EMB3D) printing, where the printed material is ink, dropped into a soft viscoelastic matrix [70]. However, EMB3D differs from SLSL in that in EMB3D, the printed part in PDMS may not be removed, whereas in SLSL, it is. Indeed, EMB3D is in essence a kind of DIW and is used for printing hydrogels inside a polymer [71] and self-assembled micro-organogels [72].

The benefit of SLSL is that it can produce circular, irregular, and complex three-dimensional channels in PDMS without the need for supports, e.g., long channels (up to 80 cm), round channels (200 µm and 500 µm in diameter), etc. [68,69,73]. There are some problems with reference to the requirements of PDMS microfluidic devices, especially R3 and R5, as discussed in Section 1 of this paper. Specifically, the shape of the channel may be of poor integrity after selectively removing the printed part. Another problem is that the optical transparency of the PDMS channel may be compromised due to the residues of the printed part in the channel. Further, the swelling and shrinkage of PDMS can be serious during the curing process. Finally, SLSL is poor in mass production.

### 3.4. Indirect PDMS Printing: Printed Mold PDMS

The printed mold approach is a kind of replica mold approach, which refers to the approach of first making a mold, and then casting a PDMS product with the mold (Figure 1d). Traditionally, the mold can be made by lithography, micro milling, and electroplating. These methods are time-consuming, labor-intensive, and usually involve expensive equipment and facilities [74]. The present paper focuses on the approach to print the mold, i.e., the printed mold approach, which may also be called the ‘master–slave approach’ in the literature [75,76,77,78,79].

The printed mold approach to PDMS microfluidic devices has four stages. Stage 1: making a mold with material X. Stage 2: casting PDMS melt into mold X. Stage 3: separating or peeling off PDMS from mold X. Stage 4: bonding the PDMS part with another part, which could be a cast PDMS part as well [80]. This paper focuses on Stages 1–3. There are several challenges with the printed mold approach to PDMS microfluidic devices to meet the requirements of microfluidic devices (see the discussion in Section 1).

The first challenge is residual stress created in the process of printing the mold. In Stage 1, the mold is subject to repeated heating and cooling, which can build up residual stress [81,82,83,84,85]. This stress can induce direct consequences such as distortion and separation of the printed part from the base plate, crack formation in a printed part, and strong anisotropic behavior in a product [86]. The residual stress can also create creep distortions, cracks, and delamination indirectly. In short, this residual stress creates a challenge to meet R3 and R4 for PDMS microfluidic devices, described in Section 1. There are several studies to reduce residual stress, e.g., heat treatment of the mold [87]. However, the heat treatment may not work for some materials [88,89], and more discussion on this point will be provided later.

The second challenge is the shrinkage of the mold during curing [90], which is related to Stage 1. The shrinkage will cause distortion of the part. In fact, shrinkage happens in any curing process (or solidification process in manufacturing in general), and therefore, it also occurs in Stage 2 (i.e., polymer casting). The shrinkage problem can be overcome by careful planning and control of the curing process [90]. It is worth mentioning that in metal casting, an effective approach to overcome shrinkage is by means of the so-called riser; however, this idea has never been tried for casting PDMS or polymers in general with a mold.

The third challenge, also related to Stage 2 (casting with PDMS), is that the PDMS resin has poor fluidity in the mold in general. This challenge is usually tackled by adding other chemicals into PDMS. However, such an approach suffers from the problem of photo-based polymerization when the curing is under some photo energy, e.g., UV light [91] and the problem of degrading the good properties of PDMS (due to the mixture of PDMS with other chemicals) for biomedical applications [22]. Another approach to improve the fluidity of PDMS is to dilute PDMS with toluene or hexane, so as to reduce the viscosity of PDMS to enable the fabrication of microfluidic devices with nanoscale holes or channels [92,93,94].

The fourth challenge, also related to Stage 3 (separation of PDMS from the mold or peeling off PDMS), is the difficulty of peeling PDMS out of the mold. Essentially, this difficulty is due to the affinity and bonding between the PDMS and the mold material. Post treatment of the mold can overcome this difficulty because it can reduce the surface roughness of the printed mold and hence ease the process of peeling off. Several post-treatment processes for the printed mold have been reported, including using solvents [9,95,96], coatings [9], sonication [9], heat treatment [90,91,97,98], UV [9,94,96,98,99,100,101,102,103], and their combination (see Table 2). There is also an approach that involves destroying the mold to get the PDMS part, e.g., dissolving the mold using water [28], chemically [34,104,105,106,107], etc. Casting of nonplanar [35] or complex shape structures of PDMS may need the destructive approach.

It can be concluded that regarding the requirements, as described in Section 1, the printed mold approach is promising to meet R1 and R4 (with the non-destructive mold approach to peel off the PDMS part). However, regarding R2 (size) and R3 (accuracy), the approach can usually only make the size of the channel ~50 μm with an accuracy of ~5 μm. The inherent problem with the printed mold approach to PDMS microfluidic devices includes (i) the shrinkage of both the mold and the PDMS casting and (ii) the affinity between the mold and the casting, which unfortunately limits the utility of this approach.

**Figure 3:**
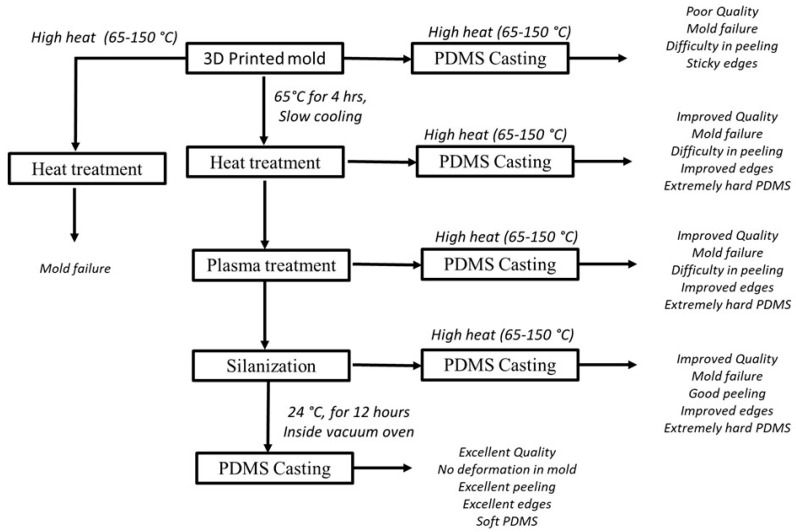
On-going work to find the optimal parameters for the post treatment of a specific mold as well as the optimal parameters for the curing of a specific PDMS.

To attempt to address the foregoing shortcomings with the printed mold approach, our group conducted a preliminary study on addressing the inherent problem (ii). Specifically, we tried to find an optimal post-treatment process for the printed mold as well as the PDMS casting such that the affinity between the mold and the PDMS part is low, thereby easing the peeling off process of the PDMS part from the mold. In our study, the mold material is ‘Full cure835 Vero white plus’ and the printing method is Polyjet 3D printer (product name: EDEN500V); see Table 1 for more information on this printer. The PDMS material is SYLGARD™ 184 Silicone Elastomer Kit (Dow Corning). The ratio of the PDMS base (Dow Corning) to the curing agent (Dow Corning) was 10:1. This mixture was degassed in a desiccator for 60 min to remove air bubbles, prior to the subsequent process, as described in Figure 3. Specifically, the process in Figure 3 has four steps: (1) heat treatment of the mold, (2) oxygen plasma treatment of the mold, (3) silanization of the mold, and (4) casting of the PDMS into the mold.

The results of our experiment are as follows: (1) the best temperature for heat treatment of the mold was found to be 65° C for 4 h, followed by overnight or slow cooling; (2) the best oxygen plasma treatment to clean the mold was found to be 50 watts, 45 millitorrs of O_2_, and a duration of 30 s; (3) silanization (wherein the sample was placed along with one drop of trichloro(1,1,2,2-perfluoocytl)silane under a vacuum chamber for 1 h) was conducted for the process of peeling off PDMS from the mold; and (4) the PDMS was cast into the mold, followed by curing the PDMS with the best parameters (curing temperature, duration, open atmosphere environment, or controlled environment) as: room temperature for curing (24 °C), duration of 24 h, a vacuum oven environment. Consequently, the PDMS casting was easily peeled off from the mold made of Full cure835 Vero white plus. However, our effort thus far is quite preliminary, and data have yet to be seriously collected, which warrants future work (see later discussions in the final section of this paper).

### 3.5. Direct Printing of PDMS

We revisit Figure 2 in the following discussion. Table 3 gives an overview of the direct printing approach to PDMS microfluidic devices, including the 3D printing method, details of which can be found in Table 1, the PDMS composite, achievements, and applications of the devices along with the references. Several remarks are further made below.

**Remark** **4.** *As mentioned before, the most salient properties with PDMS are transparency of the material with respect to light and biocompatibility. However, the worst property of PDMS with respect to 3D printing processes, especially FDM and extrusion-based methods (Table 1), is its poor printability. Therefore, for the direct printing approach to work, PDMS is always mixed with other materials to improve its printability. However, the composition can compromise the two salient properties of PDMS.*

**Remark** **5.** *The first attempted direct printing of PDMS (dissolved in hexanes) was done by creating barriers to define microchannels in a paper substrate using a modified X,Y-plotter [112]. The minimum feature size of 1 mm was achieved. Even though the resulting structure demonstrated good bending and folding properties, the overall quality of the structure was poor, i.e., structural instability and uncontrollable penetration of PDMS in the paper substrate. Another method was to use DLP techniques to create a free-standing PDMS membrane for three-dimensional gas–liquid-contactor [113]. The resist was prepared using 97.95 wt% dimethyl siloxane copolymer, 2 wt% ethyl phenyl phosphinate as a photo-initiator, and 0.05 wt% ORASOL orange dye. The dye tends to increase the resolution and prevent UV-light leaking. Cross-linking was achieved using an added photo-initiator with the help of a Hg vapor lamp at 440 nm and a brightness of 7 mW cm^−2^. The uncured resist material can be removed using isopropanol alcohol bath and ultrasonication. The concentration of the photo-initiator and the excitation dose are crucial in achieving the cross-linkage of the methacrylate functionalized PDM. The problem with this process is that the biocompatibility and transparency of the PDMS microfluidic device are significantly compromised.*

**Remark** **6.** *An ink made by mixing PDMS with platinum catalyst was used for printing complex 3D structures using full reactive inkjet printing (FRIJP) without any support [114]. During printing, the two materials were cross-linked. A controlled air environment was not required, as the reaction was not inhibited by oxygen or moisture in this process. The geometry of the resulting product depends on the mixing parameters and reaction time (the shorter the better). Mixing at elevated liquid temperature tends to produce better curing. Another process is to mix PDMS with liquid paraffin wax rapidly until it cools to room temperature [115]. Printing in this case needs to be done instantly, as the mixture has a pot life of 18 min. Curing was done at a temperature above 18 °C for 3 h. The main limitation with this process is the loss of the transparency of the final product due to the presence of wax. The printed PDMS material tends to become translucent to visible light after melting and removing the wax.*

**Remark** **7.** *Adding support material was another approach to print PDMS microfluidic devices. This process makes use of the property differences between support materials and PDMS. For instance, PDMS, which is hydrophobic in nature, is printed into a carbopol gel bath, which is hydrophilic, providing the support and dimensional stability during printing [111]. Once cured, the carbopol gel was removed by a phosphate-buffered saline solution. Lateral fusion between the extruded PDMS filaments is one of the major limitations of this process. Also, removing the support can be laborious. In addition, custom modification of the printer, print head, and tip is needed.*

**Remark** **8.** *3D printing of PDMS using the capillarity principle [54] was also reported as an alternative tool to produce soft structures. Particle suspension with capillary bridges has excellent properties such as high elastic modulus and static yield stress. In this approach, a three-part elastomeric ink was formulated, consisting of the procured PDMS microbead, uncured PDMS liquid precursor, and a water medium. A highly stretchable and elastic 3D structure was produced after cross-linking in the printed structure during heating at 85 °C. Furthermore, with such a capillary force, the method of printing PDMS into an oil-based granular gel [116] and the method of printing PDMS into an elastomer alginate hydrogel [117] were also reported in the literature. However, these processes are relatively complex along with the possible compromise of biocompatibility and transparency, and the processes are also too slow.*

**Remark** **9.** *Sylgard 184 PDMS mixed with SE 1700 PDMS elastomer seems to provide a better printability using the DLP-SLA printing technique [53,118]. SE 1700 is a shear-thinning, high-viscosity polymer, when mixed with Sylgard 184 PDMS, and demonstrates shape fidelity required for printing. A similar protocol was also recently demonstrated, replacing SE 1700 with TA (sil-thix silicone thickener–Barnes) [51]. This research demonstrated the possibility of scanning a participant’s ear using a handheld scanner, following 3D printing, which shows potential in biomedical applications. Both methods seem promising and require further investigation in relation to original properties such as transparency, biocompatibility, elasticity, etc.*

It can be concluded from the above discussion as well as the requirements set up for constructing PDMS microfluidic devices (discussed in Section 1) that (1) the state-of-the-art PDMS composite is still problematic in losing transparency and biocompatibility of the device, (2) due to the complex process in PDMS composite preparation, uncertainty in final products made by the direct printing approach is still high, (3) the shape accuracy of the microfluidic devices directly printed with PDMS composites is poor due to slow polymerization and solidification of such composites, which, unfortunately, results from the effort to improve their fluidity, and (4) the strength of the PDMS part in the printing direction is weak.

## 4. Conclusions, Future Research Directions, and Contributions

The requirements for building the PDMS microfluidic device are revised herein: (1) 3D structures with channels (R1), (2) micron scales of the channel size as well as holes (R2), (3) accuracies in the shape and geometry of channels as well as pores (about 1–5 microns) (R3), (4) proper surface features of the channel (especially for easy assembly or bonding of multiple PDMS parts) (R4), (5) mass production (R5), and (6) production rate (R6).

There are several **conclusions**, including **knowledge gaps,** with respect to the foregoing requirements, which can be drawn. First, 3D printing approaches (both direct and indirect) for fabricating PDMS microfluidic devices have the advantage over the traditional lithography approach in terms of R1 and R4, but in general, the 3D printing approach is short of meeting R2 and R3 in comparison with the traditional lithography approach. However, they have a simple set-up and relatively low costs, especially with the FDM printing method (see Table 1). In terms of R5 and R6, 3D printing approaches have a comparable performance to the traditional lithography approach. It is noted that the traditional lithography approach can only fabricate geometric features that have a limited high aspect ratio (i.e., much less than 100) and limited overall size (i.e., much less than 1000 microns). Second, among the four methods of the indirect printing approach (Figure 2), only the printed mold approach can create a full-end PDMS part, and it is most promising for the fabrication of PDMS microfluidic devices, including lab on a chip (LOC) [3,5,6] and organ on a chip (OOC) [119,120]. Specifically, the printed mold approach reduces the knowledge gap in terms of R5 and R6; further, it can produce the rounded, non-planar, sloped, and tapered cross-sectional channel in the microfluidic device, whereas the traditional lithography process can hardly do so. Third, the printed mold approach is still short of meeting R3 and R4 due to the challenges in (1) controlling the curing process of the printed mold and that of the PDMS cast with the mold, (2) bonding PDMS to PDMS in terms of alignment and bonding strength, which has not been discussed in the present paper; interested readers are directed to [80]. Fourth, the direct printing approach promises to fabricate complex PDMS microfluidic devices such as LOC and OOC, and this approach is free of the need to bond together two PDMS channels to form a hollow cross-section. However, the printability of PDMS materials remains a challenge, and it is an important factor to set up a limit in the accuracy of PDMS microfluidic devices by the direct printing approach. To tackle this challenge, the popular idea is to mix additives with PDMS, but this can unfortunately compromise the biocompatibility and optical transparency of the PDMS mixture. Another idea to tackle the challenge is to replace PDMS with other materials that have similar properties to PDMS but good printability. Nevertheless, this idea is premature.

Therefore, **future work** toward closing the knowledge gaps is warranted. First, post treatment of the printed mold needs attention, especially to continue the on-going effort in our group, presented in Section 3.4, to understand the mechanism of the excellent result achieved. It is worth mentioning that the post treatment of the mold is a crucial factor for fabrication of PDMS microfluidic devices to meet R1–R4 with even better results than the traditional lithography approach. Second, some surface features, such as roughness and dimension control, grain size, crack length/width, and surface stress, need to be examined for their possible effect on meeting R4. Indeed, these surface features can affect the bonding strength for two layers of PDMS being bonded or a PDMS layer being bonded to a different substrate, e.g., glass, silicon etc. [121]. In future work, the micro-CT technique may be employed to examine details of the interface of the PDMS casting and the mold, and details of the bonding interface between one PDMS and another PDMS or other different materials should be explored, because this technique is non-destructive and in situ and therefore allows us to gain accurate information. It is noted that this technique is not expected to replace instruments such as atomic force microscopy (AFM) and scanning electron microscopy (SEM). Third, properties such as opacity, refraction index, hydrophobicity, and those related to biochemical behavior of PDMS (fabricated using the printed mold approach) need to be analyzed in terms of how well and why R2–R4 are satisfied [122]. Fourth, the structure of an OOC made of PDMS needs to be studied for being printable or improving printability with the direct printing approach against the requirements of microfluidic devices (R1–R6). The development of OOCs requires creating complex 3D deformable structures of the device such that the device can sense and respond to the change in biological activities of cells [3]. Fifth, the direct printing of PDMS microfluidic devices warrants further studies, especially with additives to PDMS to improve its printability and thus to improve the quality of devices in terms of R1, R2, and R3. Sixth, the combination of different AM processes, e.g., twin photon printing (TPP) [123,124] and stereo-lithography processes, is worth studying, because the former can build nanometer features but with a limited area, while the latter can build micron or millimeter features over a large area; the best combination is also called hybridization [125]. This future work is expected to fabricate PDMS microfluidic devices to meet R1–R3 better. As a general note, in the future, additional properties or behaviors such as reliability, robustness, and resilience [126,127,128] of a PDMS microfluidic device will be studied. Such a microfluidic device may look like a robot [3], especially an adjustable and reconfigurable robot [129,130]. Finally, printing of microfluidic devices, made of PDMS along with one or more other materials, is an interesting topic, as a more sophisticated PDMS-based microfluidic device can be built. It is worth mentioning that the literature [131,132,133] on multi-material printing is helpful to print such microfluidic devices.

Several recently published review papers are relevant to the fabrication of PDMS microfluidic devices. The focus of [134] is on the direct printing approach; specifically, several direct printing methods are discussed. As the paper was published in 2016, the most recent processes on the direct printing approach are missed. Further, in [134], the printed mold method, a kind of the indirect printing approach (as we defined in this paper), was discussed, missing the recent indirect printing methods, including the micro contact printing, printing inside PDMS, and printing on top of PDMS (see Figure 1). The focus of [135] is on the direct printing approach using AM processes and selection of their process parameters. First, this focus is relatively narrow. Second, several AM processes, as classified in our paper (Table 1), were missed in [126]. Therefore, the review in [126] is neither comprehensive nor complete. The focus of [136] is on the printed mold approach, as classified in our paper. The shortcoming of [136] is that the paper only vaguely and generally discusses knowledge gaps. Moreover, several AM processes against the classification of AMs (see Table 1) were missed in [127]. Further, the paper does not have any division of the whole process into four stages, as defined in our paper, nor a comprehensive discussion of challenges at each of the stages.

Our paper makes several contributions. First, in the sub-field of the 3D printing of PDMS microfluidic devices, this paper has provided a classification of the processes involving 3D printing for PDMS microfluidic devices. Second, in the field of 3D printing, this paper has provided a unique view, i.e., design thinking, to render a comprehensive classification of 3D printing technology. Third, this paper has clarified some confusion in terminology or concepts in the current literature, namely (1) the relationship of AM and 3D printing, where 3D printing is a kind of AM, but is also a nickname for all AM processes; and (2) the relationships among the concepts such as lithography, soft lithography, printed mold, replica mold, and master–slave.

## Figures and Tables

**Table 1:** Classification of the AM processes.

Starting Status of Material	Principle of Sub-Process 1 for F1	Principle of Sub-Process 2 for F2	Selected Materials	Name
Polymer melt or resin	Melt solidification	Fusion and diffusion	Polycarbonate (PC), acrylonitrile butadiene styrene (ABS), polylactic acid (PLA), polyetherimide (ULTEM), nylon, carbon-filled nylon, acrylonitrile styrene acrylate (ASA)	Fused deposition modeling (FDM), 3D printing (nickname)
Solid (sheet)	Laser cutting of sheet	Diffusion	Adhesive-coated paper, plastic, or metal laminates	Laminated object manufacturing (LOM)
	Laser cutting of sheet	Diffusion and glue	Polyamides (PA), polystyrenes (PS), thermoplastic elastomers (TPE), polyaryletherketones (PAEK).	Selective deposition lamination (SDL)
	Mechanical cutting of sheet	Diffusion under ultrasonic pressure	Various aluminum alloys, nickel alloys, brass, and steels, etc.	Ultrasonic additive manufacturing (UAM)
Solid (powder)	Powder glued with binder	Diffusion and gluing	ABS, ASA, PLA, polycaprolactone (PCL), Vero	Binder jetting
	Thermal diffusion	Thermal diffusion	PCL, PLA, metal	Selective laser sintering (SLS)
Liquid	Photo-resistivity	Photo-resistivity	Thermosetting acrylates and epoxy	Stereolithography (SLA)
	Photo-resistivity	Photo-resistivity	405 nm clear resin (Anycubic)	Digital light processing (DLP)
	Liquid solidification	Diffusion	Thermosetting resin with adequate viscosity	Direct writing (DW) or direct ink writing (DIW)
	Droplet/photo-resistivity	Photo-resistivity	Vero white, rubber, polypropylene	Polyjet
	Extrusion/microwave irradiation or heating	Microwave irradiation or heating	Concrete, ceramics, wood, clay, food products, biomaterials, silicon, polyurethane (PU)	Liquid deposition modeling (LDM)
	Droplet/extrusion	Thermal diffusion or UV curing	Hydrogels, bio-compatible copolymers, and cell spheroid binders and powders, polymers, and small molecules	Inkjet bioprinter and material extrusion bioprinter
	Extrusion	Thermal diffusion or UV curing	Low-viscosity resins, poly(ε-caprolactone) (PCL), silver paste	E-Jet printing and Electric-Field-Driven (EFD) printing

**Table 2:** The post-treatment process for the printed mold along with the conditions for PDMS curing.

Type of Printing	Material of Printing	Treatment				PDMS Curing	Ref.
		1	2	3	4		
SLA	PIC100	Flashlight polymerization	Ethanol (100%) rinse at 37 °C for 7 h			Overnight at 60 °C	[95]
Micro-SLA	Proprietary	Sonicated in ethanol for 2 min	Ink (Pentel NN60) airbrushing			65 °C for 2 h	[9]
SLA		Ethanol rinse for 1 min, air dry, UV cured for 600 s	130 °C for 4 h in oven	Oxygen plasma at high for 3 min	Silanization for 30 min	80 °C for 4 h	[108]
SLA		Ethanol wash, air dry	UV cure	80 °C for 24 h		80 °C for 4 h	[99]
DLP-SLA	BV-003	5 min UV	Isopropanol for 6 h	Corona treatment high power and atm pressure for 1 min	Silanization for 3 h	70 °C for 2 h	[100]
FDM	PLA	12 h at 60 °C				48 h at room temperature (~25 °C)	[97]
PolyJet		Baked overnight at 90 °C					[109]

**Table 3:** Direct printing methods for PDMS microfluidic devices.

Method of Printing	Printing Material	Achievement	Limitation	Application and Reference
Custom made microextrusion-based 3D printer (μE-3DP)	PDMS-TA (sil-thix silicone thickener–Barnes)	Demonstrated possibility of printing ear and compression stain evaluated. Needs further investigation	Optical transparency is questionable; printing accuracy not evaluated; biocompatibility not evaluated	Facial prosthesis [51]
Liquid dispenser (Pandasky OL-D331)	PDMS diluted in acetone	PDMS barriers printed using a liquid dispenser	Flow barrier in the form of filter paper is required	Microfluidics [52]
Drop-on-demand (DOD) piezoelectric inkjet printing	PDMS- decane–toluene ink	Improved satellite effect problem	Toluene is hazardous. Biocompatibility issues	Flexible wearable electronics [50]
Extrusion through nozzle (custom 3D printer modified from CNC setup)	PDMS ink (precured PDMS microbeads, uncured PDMS liquid precursor, and water medium)	Can be 3D printed and cured both in air and under water; bio scaffolds on live tissue	Optical transparency is questionable	[54]
Electric-field-driven (EFD) microscale 3D printing (EM3DP-2A)	PDMS Insufficient data	PDMS and curing agent passive mixing at the source using microfluidic chip; no need for vacuum defoaming	High voltage: air flow and curing temperature and printing speed are key parameters	Microlens array, [56]
Selective laser sintering (SLS)	Dynamic covalent cross-linked PDMS (PDMS CANs)	Properties not evaluated. Printing accuracy not evaluated	Properties and printing accuracy need to be evaluated	Sportswear insole [48]
UV LED DLP stereolithography (Asiga Freeform PRO2 printer)	PDMS–thiourea based resins	Noncytotoxic; tunable mechanical properties; plastic deformation, highly recoverable	Optical transparency is questionable	Soft robotics, medical devices [44]
DLP–SLA system (Asiga MAX X27 UV printer)	Photoreactive methacrylate–PDMS copolymer of (98.6%) photoinitiator TPO-L (0.8%); photosensitizer ITX (0.4%); photo absorber and Sudan I (0.2%)	60 μm deep channels and 20 μm thick membranes produced; gas-permeable; transparency issues; lower transmission	Difficult to remove unpolymerized resin from the micron-scale channels due to high viscosity of the resin. Young’s modulus is much larger and elongation at break is much smaller; not suitable for pneumatic pump application	Microfluidics [45]
Customized ultraviolet-assisted direct ink writing (UV-DIW) 3D printer	pPDMS+ M-PDMS + TPO-L ink	Excellent mechanical properties; optical transparency close to Sylgard 184 PDMS	3D printing followed by thermal cross linking. Biocompatibility needs further evaluation.	Microfluidics, flexible electronics [47]
Electrohydrodynamic (EHD) inkjet printing	PDMS–toluene (toluene has a lower viscosity and better volatility)	Printing accuracy not evaluated	Toluene is hazardous; biocompatibility issues	Network structure [55]
FDM (3DPRN LAB 3D)	PDMS–Na–CMC composite	Both Neat PDMS and PDMS composite filaments are made and printed; well-adherent layers of composite material	Irregular samples; no control of geometry; no satisfying results have been obtained	[41]
UV LED DLP INKREDIBLE 3D bioprinter (Cellink, Sweden)	Blends of two PDMS elastomers, SE 1700 and Sylgard 184	Improved three-fold mechanical properties with regard to casting mold due to decreased porosity of bubble entrapment.	Printing accuracy not evaluated	Cell adhesion studies [110]
Extrusion through nozzle (home-made 3D printer)	Blends of two PDMS elastomers, SE 1700 and Sylgard 184	Super hydrophobicity; porous structure	Extremally dependent on printing speed; optical transparency is questionable; printing accuracy not evaluated	Super hydrophobic porous film [53]
FDM (replicator 2 3D printer (MakerBot))	PDMS ink and carbopol support bath	Carbopol supports curing times up to 72 h	Releasing printed PDMS from the carbopol support is tedious; cross-section morphology needs to be improved.	[111]
UV LED DLP stereolithography	3DP-PDMS resin	Transparent; cytocompatible; gas-permeable; highly elastic	Low Young’s modulus; ~500 μm resolution achievable with unsupported structure	Microfluidics [43]
Inkjet printer (Fujifilm Dimatix DMP)	1. PDMS mixed with isobutyl acetate (IBA) solvent 2. PDMS mixed with octyl acetate (OA) solvent	Final printed and cured PDMS–OA is free of solvent residues and resembles traditional casting; 5 μm thickness in each layer and maximum of 8 layers possible.	Ink cartridge shelf life 2 days; needs to be stored inside refrigerator. Nozzles clog quickly, IBA- non-reliable, and difficulty in reproducible jetting.; IBA- hazardous; OA slower evaporating	Soft electrical applications [49]
UV curing (same parameters as SLA)	PDMS-(trimethyl)pentamethylcyclopentadienylplatinum(IV) (Cp*PtMe3) catalyst	Good tensile strength, PDMS formulation exhibits very good aging properties over time.	UV-exposed samples seem yellowed. Under-cured material with UV alone; requires further UV or thermal curing. Finally cured after few weeks	[46]

## Data Availability

Data will be made available based on request.
